# Comprehensive profiling of zebrafish hepatic proximal promoter CpG island methylation and its modification during chemical carcinogenesis

**DOI:** 10.1186/1471-2164-12-3

**Published:** 2011-01-04

**Authors:** Leda Mirbahai, Timothy D Williams, Huiqing Zhan, Zhiyuan Gong, J Kevin Chipman

**Affiliations:** 1 School of Biosciences, The University of Birmingham, Edgbaston, Birmingham, B15 2TT, UK; 2 Department of Biological Sciences, 14 Science Drive 4, National University of Singapore, Singapore 117543

## Abstract

**Background:**

DNA methylation is an epigenetic mechanism associated with regulation of gene expression and it is modulated during chemical carcinogenesis. The zebrafish is increasingly employed as a human disease model; however there is a lack of information on DNA methylation in zebrafish and during fish tumorigenesis.

**Results:**

A novel CpG island tiling array containing 44,000 probes, in combination with immunoprecipitation of methylated DNA, was used to achieve the first comprehensive methylation profiling of normal adult zebrafish liver. DNA methylation alterations were detected in zebrafish liver tumors induced by the environmental carcinogen 7, 12-dimethylbenz(a)anthracene. Genes significantly hypomethylated in tumors were associated particularly with proliferation, glycolysis, transcription, cell cycle, apoptosis, growth and metastasis. Hypermethylated genes included those associated with anti-angiogenesis and cellular adhesion. Of 49 genes that were altered in expression within tumors, and which also had appropriate CpG islands and were co-represented on the tiling array, approximately 45% showed significant changes in both gene expression and methylation.

**Conclusion:**

The functional pathways containing differentially methylated genes in zebrafish hepatocellular carcinoma have also been reported to be aberrantly methylated during tumorigenesis in humans. These findings increase the confidence in the use of zebrafish as a model for human cancer in addition to providing the first comprehensive mapping of DNA methylation in the normal adult zebrafish liver.

## Background

Tumorigenesis by chemical carcinogens is a multistep process [1] with accumulation of both mutations and epigenetic aberrations in regulatory regions of genes and disruption of cellular signaling pathways [2,3]. In particular, DNA methylation at CpG dinucleotides is an important component of epigenetic gene expression regulation [4], resulting in modulation of protein-DNA interactions [5,6]. Aberrant methylation of CpG islands (CGI) in the promoter and exonic regions [7,8], and changes in gene expression, have been associated with tumorigenesis [7,9,10]. Global hypomethylation occurs in most human tumors [3,4,7] leading to potential activation of imprinted genes, parasite sequences and oncogenes and increased chromosome instability [3]. In addition, hypermethylation of genes associated with negative regulation of tumorigenesis, such as tumor suppressor genes (TSG), DNA repair genes, and anti-angiogenic genes, is a common and key characteristic of neoplastic cells [4,10-13]. Furthermore, a range of rodent carcinogens alter methylation status contributing to the carcinogenic mechanisms [14].

Fish have been used as models to study tumors induced by environmental carcinogens. For example, rainbow trout (*Oncorhynchus mykiss*) [15], zebrafish (*Danio rerio*) [16], guppy (*Poecilia reticulata*) [17], platyfish (*Xiphophorus sp*.) [18] and medaka (*Oryzias latipes*) [17] have been employed in carcinogen bioassays. Zebrafish is a particularly well established model for investigating embryogenesis, organogenesis, environmental carcinogenesis and for modeling human diseases such as cancer [15,19-23]. Chemically-induced tumors in zebrafish and tumors in humans are histopathologically very similar [20,24] and orthologous TSGs and oncogenes in human and fish have been identified [20]. Recent studies comparing hepatic gene expression in human and zebrafish tumors demonstrated conservation of gene expression profiles at different stages of tumor aggressiveness between these two phylogenetically distant species [23]. However, the contribution of altered methylation to such changes is unknown.

Although there are reports on global levels of methylation during embryogenesis or in adults [25-27], effect of temperature on global levels of methylation [28] methylation profiling in zebrafish embryo [29] and role of chromatin mediated gene regulation during embryogenesis [19], to our knowledge no study has been published on the DNA methylation patterns or methylation changes associated with carcinogenesis in adult zebrafish. Therefore, the aims of the study were to achieve a comprehensive mapping of zebrafish hepatic proximal promoter CGI methylation in both normal liver and in chemically induced hepatocellular carcinoma (HCC) tumors. We aimed to determine if there was a linkage between methylation and the observed changes in the zebrafish HCC gene expression, and to compare biological pathways represented by altered gene methylation in zebrafish HCC with pathways commonly altered in human HCC.

To our knowledge this paper is the first to describe methyl-DNA immunoprecipitation (MeDIP) in combination with a CGI zebrafish tiling array for establishing normal and HCC methylation profiles in the liver of any adult fish species. This microarray, in combination with well-established methylation immunoprecipitation, serves as a powerful tool for elucidating comprehensive methylation profiles. To further validate the data derived from the tiling array, bisulfite sequencing PCR was used.

## Results

### Global measurement of DNA methylation

**Figure 1:**
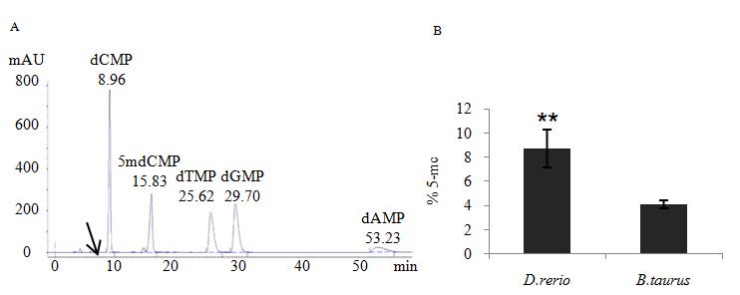
**Measurement of global percentage of methylated DNA in zebrafish and calf using HPLC**. A. Determination of the retention time of five standard mononucleotides using HPLC. An arrow indicates absence of uracil peak showing that the sample is not contaminated with RNA. B. Percentage of DNA methylated in healthy zebrafish (*D.rerio*) and calf (*B.taurus*) thymus. Global percentage of methylated DNA in zebrafish is 2.14 fold higher than calf. Data are the mean ± SEM of three independent experiments. ** Significantly different from calf (p < 0.01).

To establish the global level of cytosine methylation in zebrafish liver in comparison to mammals, reverse-phase high performance liquid chromatography (HPLC) was performed (Figure 1A). Absence of RNA contamination was confirmed using uracil as a reference material. As shown in Figure 1B, zebrafish liver has a statistically significant 2.14 fold higher methylation level (p-value <0.01) than that of calf (*Bos taurus*) thymus. This is in agreement with previous published data showing higher levels of DNA methylation in fish compared to mammals [27].

### Unbiased enrichment of methylated DNA using methyl-DNA immunoprecipitation (MeDIP)

To optimize and test the specificity of the immunoprecipitation method, CGI regions of 14 genes (15 regions) located 1.5 kb upstream to 1 kb downstream of transcriptional start sites (TSS) were screened for methylation of cytosine using bisulfite sequencing PCR (Additional file 1, Table S1). Following bisulfite treatment and sequencing of the 14 genes, CGIs located at the promoter region of the "*no-tail*" (*ntla*) gene were found to be completely methylated while the remaining 13 genes, including *glutathione S- transferase P1 *(*gstp1*), were completely un-methylated (Additional file 2, Figures S1 and S2). The *no-tail *gene and the *glutathione S-transferase P1 *gene were thus used as fully methylated (positive control) and un-methylated (negative control) DNA regions for validating the immunoprecipitation enrichment method.

DNA was immunoprecipitated and bisulfite-treated. Equal amounts of immunoprecipitated, bisulfite-treated DNA were amplified for the two genes of interest. The gel images achieved indicated a clear enrichment of methylated *ntla *gene in comparison to un-methylated *glutathione S-transferase P1 *using 5-methyl cytosine antibody (the primers used and the regions amplified are shown in Additional file 3, Table S1 and the gel electrophoresis image is shown in Additional file 4, Figure S1).

### Design of CGI (1.5 kb upstream to 1 kb downstream of TSS) zebrafish tiling array and comprehensive mapping of adult zebrafish liver

**Figure 2:**
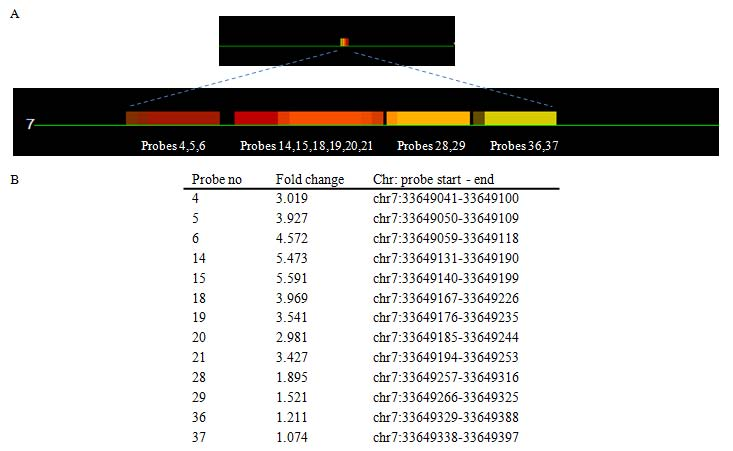
**Chromosomal mapping of the probes**. A. All probes were mapped onto zebrafish chromosomes. As an example, probes for *coronin, actin binding protein 2ba *gene (*coro2ba*; ENSDARG00000079440) on chromosome 7 are shown. A CGI was predicted at the region between 33649014 and 33649398. Colors indicate the intensity of the probe fluorescent signal (red: hypermethylation, yellow: no change, blue: hypomethylation). B. The mean fold changes between control and tumor samples for each probe are shown.

**Figure 3:**
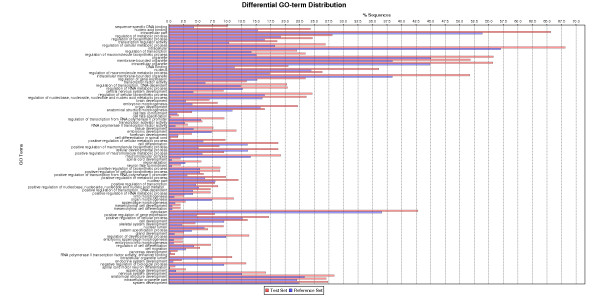
**Gene ontology (GO) terms significantly over-represented in the genes with DNA methylation levels 2 fold below the median level in zebrafish healthy liver i.e. lower methylation level (FDR< 5%)**. These data are derived from those shown in Additional file 6, Table S4 which displays the mean normalized fluorescence intensity for 4 healthy zebrafish liver samples for all probes that passed filtering steps.

**Figure 4:**
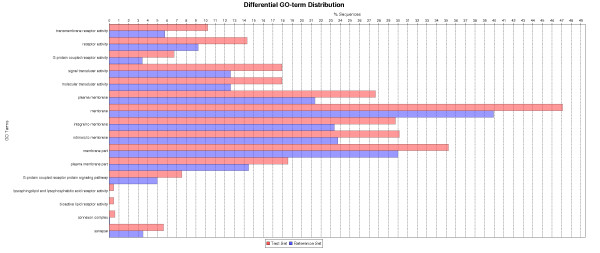
**Gene ontology (GO) terms significantly over-represented in the genes with DNA methylation levels 2 fold above the median level in zebrafish healthy liver i.e. higher methylation level (FDR< 5%)**. These data are derived from those shown in Additional file 6, Table S4 which displays the mean normalized fluorescence intensity for 4 healthy zebrafish liver samples for all probes that passed filtering steps.

Probes (43,960) were designed to cover the CGIs located 1.5 kb upstream to 1 kb downstream of the TSS of 6,024 genes. A list of genes with predicted CGIs, number of CGIs for each chromosome and location on the chromosome are presented in Additional file 5, Table S1. Where possible, 60-mer probes were tiled over the CGIs with 25 bp spacing, covering the entire region (Figure 2). The negative control consisted of PCR amplified genomic DNA, immunoprecipitated and labeled with Cy-5. As methylation is removed during amplification little DNA was precipitated as expected. A uniform low intensity signal was measured for all probes represented on the array (Additional file 6, Table S1) representing non-specific binding of DNA to the 5-methylcytosine antibody. The positive control was achieved by treatment of DNA with CpG methyltransferase (*Sss I*) in the presence of *S*-adenosylmethionine (SAM) prior to MeDIP. This resulted in complete methylation of all CGIs and detection of a uniformly high fluorescent signal for all probes (Additional file 6, Table S1). Using our CGI tiling array we achieved a comprehensive mapping of the DNA methylation at CGIs in adult zebrafish liver at the 1.5 kb upstream to 1 kb downstream of gene TSS. The list of all genes on the tiling array and their methylation levels are presented in Additional file 6, Table S4. Those genes with values 2 fold above the median level (higher methylation) and 2 fold below the median level (lower methylation) in control are highlighted. Gene ontology analysis using Blast2GO identified GO terms significantly (False Discovery Rate (FDR) <5%) over-represented amongst lower- and higher-methylated genes in control samples. For lower methylated genes, the GO terms were related particularly to involvement in hormone secretion, cellular response to hormone stimulation, regulation of transcription, sex determination (*dmrt*), and regulation of apoptosis (Figure 3). Higher methylated genes included those involved in molecular transducer activity and the connexin complex (Figure 4).

### Methylation analysis of zebrafish hepatocellular carcinoma and comparison to gene expression

**Figure 5:**
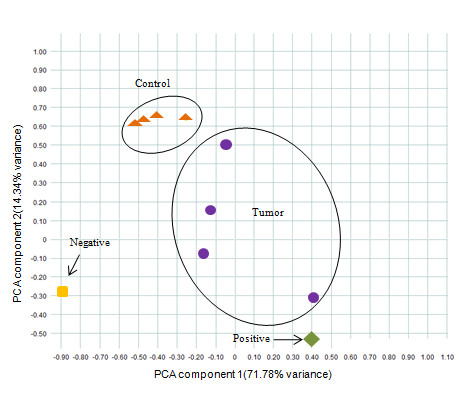
**Principal component analysis (PCA) scores plot of DNA methylation data**. Zebrafish HCC samples (purple), healthy zebrafish liver samples (orange), positive control of artificially methylated genomic DNA (green) and negative control of artificially un-methylated genomic DNA (yellow) were separated based on treatment along the PC1 and PC2 axes.

**Figure 6:**
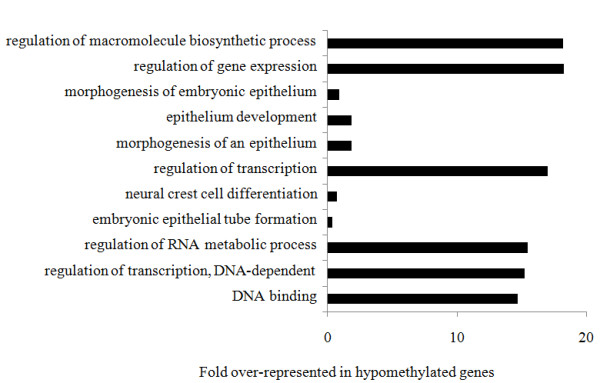
**Gene ontology (GO) terms significantly over-represented in the list of genes hypomethylated in zebrafish HCC compared to control (FDR< 10%)**.

DNA extracted from zebrafish HCC was processed by MeDIP, labeled with fluorescent Cy5-dCTP and hybridized to the tiling array against input genomic DNA. Following quality checks and normalization, probes were mapped onto the chromosomes and two lists, containing hypomethylated and hypermethylated regions in comparison to control, were generated (fold change >1.5, p-value <0.05). It was apparent that most differentially methylated regions in HCC were hypomethylated (712 probes) in comparison to 168 hypermethylated regions. Examples of genes relevant to cancer that contained aberrant methylation are shown in Table 1 (the full list of genes and gene ontology analyses are presented in Additional file 6, Tables S2 and S3). Using a principal components analysis (PCA) scores plot of differentially methylated regions, the four groups of HCC, controls, positive and negative control were separated based on treatment along the PC1 and PC2 axes (Figure 5). Gene ontology analysis using Blast2GO was carried out to find changes in GO term representation in lists of hypomethylated and hypermethylated genes. Functionally related genes with statistically significant differential methylation between tumors and controls are presented in Figure 6 (False Discovery Rate (FDR) <10%).

**Table 1:** Identified genes with altered methylation in zebrafish HCC.

Gene name	Gene ID	Chr. no	CGI region
**A. Hypomethylated Genes**			
**Proliferation**			
Kruppel-like factor 12b	ENSDARG00000032197	9	31117162 - 31117483
Insulin-like growth factor binding protein 5a	ENSDARG00000025348	9	49194032 - 49194791
Insulin-like growth factor binding protein 2a	ENSDARG00000031422	9	49290381 - 49290816
Insulin-like growth factor binding protein 1b	ENSDARG00000038666	2	178184 - 178306
Insulin-like growth factor-binding protein 2A precursor	ENSDARG00000052470	6	22745784 - 22746301
Estrogen related receptor delta fragment	ENSDARG00000015064	18	48225445 - 48225631
Hypothetical protein LOC550398	ENSDARG00000043587	1	51038733 - 51038845
**Stress**			
Similar to SH2 domain containing 3C	ENSDARG00000028099	10	14303115 - 14303263
PI-kinase-related SMG-1	ENSDARG00000054570	3	28544220 - 28544532
**Glycolysis**			
Enolase 2	ENSDARG00000014287	19	4698036 - 4698168
Hexokinase 1	ENSDARG00000039452	13	23684991 - 23685203
**Cell cycle, metastasis, adhesion, cell growth, stress**			
c-Jun protein	ENSDARG00000043531	20	14274343 - 14274635
BCL2-associated athanogene 5	ENSDARG00000017316	13	17324708 - 17324877
Angiopoietin-like 3	ENSDARG00000044365	6	34165085 - 34165237
Angiopoietin-1 receptor precursor	ENSDARG00000028663	5	625275 - 625436
Ras homolog gene family, member Ua	ENSDARG00000019709	13	25136705 - 25136882
Menage a trois homolog 1	ENSDARG00000002077	13	31141636 - 31142019
Serine/threonine and tyrosine protein kinase	ENSDARG00000000853	22	461194 - 461772
**DNA binding and regulation of transcription**			
DNA (cytosine-5-)-methyltransferase 6	ENSDARG00000015566	17	34759411 - 34759539
Leucine zipper protein 2 precursor	ENSDARG00000068247	18	35625841 - 35625986
Histone H2A	ENSDARG00000001915	1	724191 - 724473
Histone deacetylase 4	ENSDARG00000041204	9	46375505 - 46375895
Homeobox protein Hox-B5a	ENSDARG00000013057	3	20707021 - 20707754
Pancreas transcription factor 1 subunit alpha	ENSDARG00000014479	2	27672474 - 27672641
Hypothetical protein LOC692291	ENSDARG00000012833	17	12689887 - 12690054
Metastasis associated 1 family, member 2	ENSDARG00000013031	7	17243535 - 17244290
Homeobox protein Hox-B4a	ENSDARG00000013533	3	20721064 - 20721722
Lysine-specific demethylase 4A	ENSDARG00000018782	6	4575451 - 4575994
RAB11 family interacting protein 4 (class II) a	ENSDARG00000053855	25	13380260 - 13380369
			
**B. Hypermethylated Genes**			
**Anti-angiogenesis**			
Hematopoietically-expressed homeobox protein hhex	ENSDARG00000074250	12	43558447 - 43558684
**Cell-cell adhesion**			
Hypothetical protein LOC678612	ENSDARG00000069505	21	27963202 - 27963305
Novel protocadherin protein fragment	ENSDARG00000053462	1	55112983 - 55113251
**Transporter**			
ATP-binding cassette, sub-family A, member 5	ENSDARG00000074041	12	38940033 - 38940251
**immune system**			
Novel protein similar to nuclear factor, interleukin 3 regulated	ENSDARG00000071398	22	21400978 - 21401243
Novel protein fragment	ENSDARG00000053462	1	55112983 - 55113251
C5a anaphylatoxin chemotactic receptor	ENSDARG00000040319	18	45692130 - 45692420
**Angiogenesis and oxidative stress protection**			
Vascular endothelial zinc finger 1	ENSDARG00000008247	10	35492470 - 35493037
**Membrane**			
Coronin, actin binding protein 2ba	ENSDARG00000079440	7	33649014 - 33649398

A. Genes identified as hypomethylated in HCC as measured by CGI-tiling array (1.5-fold less methylation in tumor samples than controls, P-value < 0.05). B. Genes identified as hypermethylated in HCC as measured by CGI-tiling array (1.5-fold more methylation in tumor samples than controls, P-value < 0.05). Full data is shown in Additional file 6, Tables S2 and S3.

**Figure 7:**
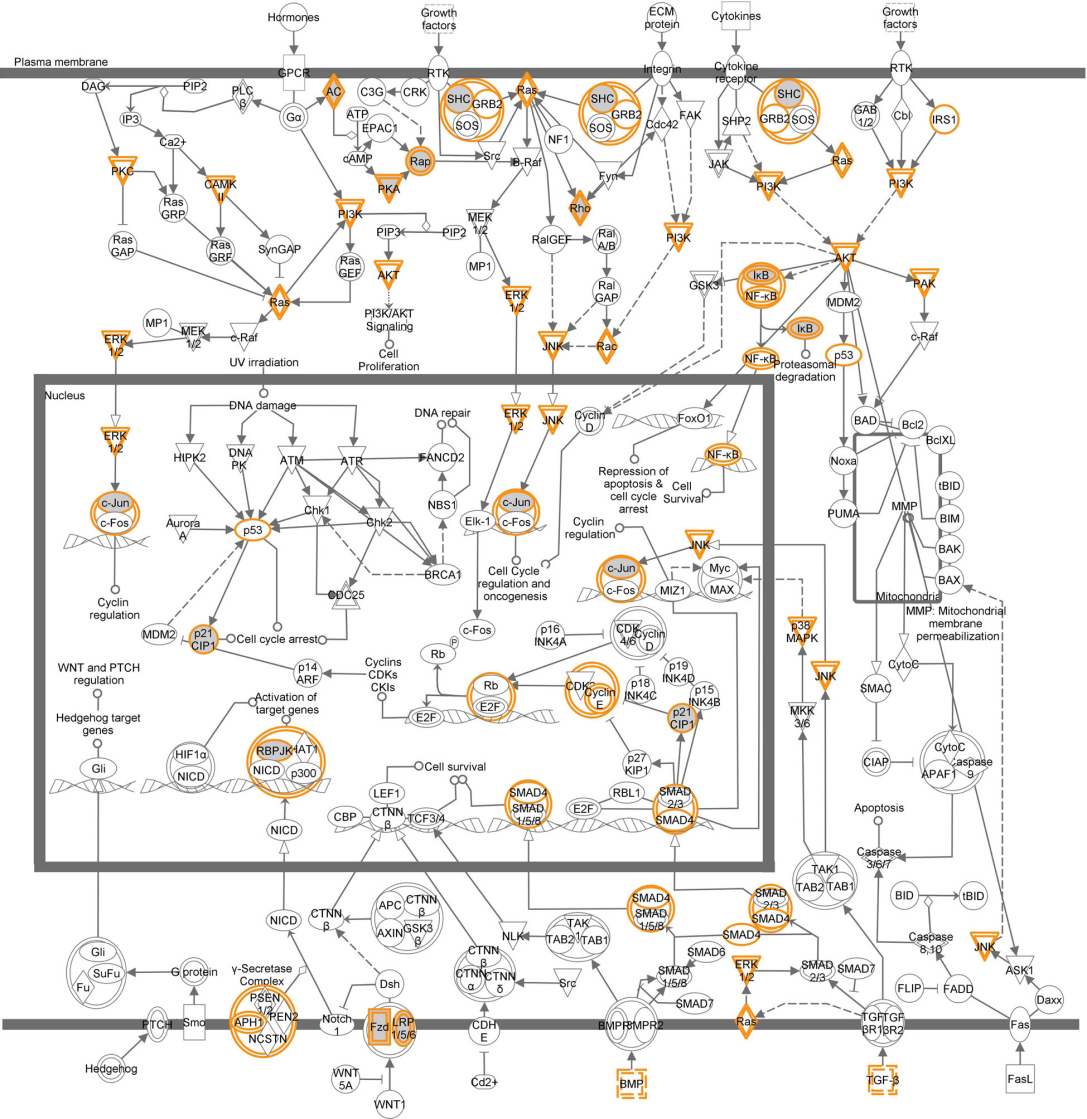
**Biological network of genes linked to the canonical pathway "molecular mechanisms of cancer" that were hypomethylated (fold change >1.5) in zebrafish hepatocellular carcinoma compared to healthy liver**. This diagram shows the genes that were hypomethylated in zebrafish HCC with grey shading. Additionally, orange outlines indicate the molecules associated with the hypomethylated genes via Ingenuity pathways. Direct interactions are shown as solid lines and indirect as dashed lines. Biological network analysis was performed using Ingenuity Pathway Analysis.

In addition to Blast2GO, Ingenuity Pathway Analysis (IPA) was performed to characterize the functional relationships between genes that were altered in methylation in tumors compared to control. Figure 7 illustrates hypomethylated genes in zebrafish tumors (fold change >1.5) related to the canonical pathway "molecular mechanisms of cancer" in humans these included *c-jun, shc and p21*.

IPA identified significant networks, top functions and canonical pathways associated with the differentially methylated genes for each comparison analyzed. Significantly over represented (FDR <5%) categories of functions for both hypo- and hyper-methylated genes in zebrafish HCC are shown in Additional file 7, tables S1 and S2.

**Figure 8:**
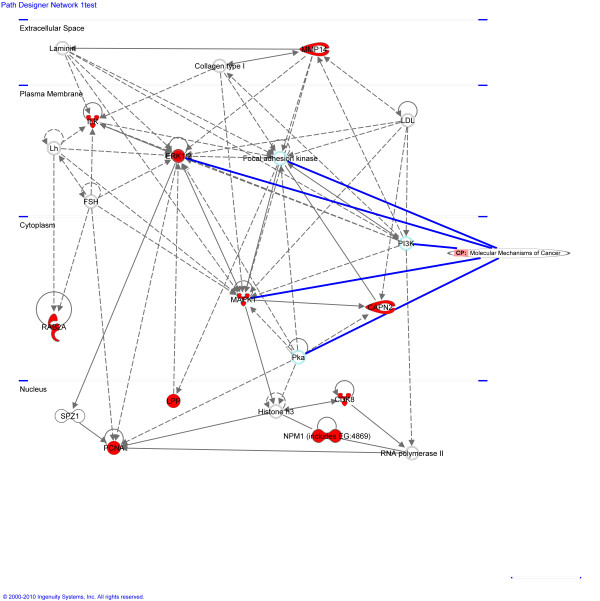
**Ingenuity network predicted for genes that were both hypomethylated and increased in expression in zebrafish HCC (shaded red)**. In this diagram molecules that are part of the canonical pathway "molecular mechanisms of cancer" are indicated with a blue line.

In a previous study [23], gene expression changes in zebrafish HCC produced by the same chemical treatment were profiled. We used data from this study to compare alteration in gene expression with DNA methylation in HCC samples. Comparisons are limited to the genes represented on both the expression array and the CGI tiling array. Expression of 194 genes that significantly differed between healthy and zebrafish HCC were investigated for methylation. From the 194 genes with altered gene expression, 68 genes had CGIs at the 1.5 kb upstream to 1 kb downstream of TSS. Due to the criteria used for designing probes, only 49 genes from the previously identified 68 genes were represented on the tiling array and were investigated for comparison of DNA methylation level with gene expression. In total 22 genes were identified showing both significantly altered transcription and methylation (> 1.5 fold change) in zebrafish HCC (Table 2, A full list of the 194 genes investigated is presented in Additional file 8, Table S1). Most genes identified were hypomethylated with up-regulated gene expression, such as *mitogen-activated protein kinase 1 *(*mapk1*), *cell division protein kinase 8 *(*cdk8*), *RAB2A, member ras oncogene family *(*rab2*) and *proliferating cell nuclear antigen *(*pcna*). IPA was applied to the list of genes that were significantly hypomethylated with up-regulated gene expression. The networks highlighted (Figure 8) contained molecules involved in formation of cancer, proliferation and transformation of cells in humans. For example, *pcna *is involved in formation of the replication fork and directs maintenance methyltransferases to the newly synthesized DNA strand. *Mapk1*, *capn2 *and *erk *were additional molecules linked with human tumorigenesis.

**Table 2:** Comparison of DNA methylation levels with gene expression levels.

Gene name	Symbol	Chr. no	Expression level	Methylation level	Gene ID
Matrix metalloproteinase 14 (membrane-inserted) alpha	*mmp14a*	7	↑	↓	ENSDARG00000002235
Histidyl-tRNA synthetase	*hars*	14	↑	↓	ENSDARG00000003693
Tubulin, alpha 8 like 4	*tuba8l4*	6	↑	↓	ENSDARG00000006260
E2F transcription factor 6	*e2f6*	20	↓	↓	ENSDARG00000008119
Acetoacetyl-CoA synthetase	*aacs*	5	↑	↑	ENSDARG00000012468
Nucleophosmin 1	*npm1*	10	↑	↓	ENSDARG00000014329
Cell division protein kinase 8 (probe 54)	*cdk8*	24	↑	↓	ENSDARG00000016496
Cell division protein kinase 8 (probes 21, 31)	*cdk8*	24	↑	↑	ENSDARG00000016496
Hepatoma-derived growth factor-related protein 2	*hdgfrp2*	22	↑	↓	ENSDARG00000019530
RAB2A, member RAS oncogene family	*rab2*	2	↑	↓	ENSDARG00000020261
LIM domain containing preferred translocation partner in lipoma	*lpp*	6	↑	↓	ENSDARG00000023578
Insulin-like growth factor binding protein 5a	*igfbp5b*	9	↓	↓	ENSDARG00000025348
Mitogen-activated protein kinase 1	*mapk1*	5	↑	↓	ENSDARG00000027552
Cancer susceptibility candidate gene 3 protein homolog	*casc3*	3	↑	↑	ENSDARG00000029911
Hnrpa0l protein	*hnrpa0l*	14	↑	↓	ENSDARG00000036161
Cyclin T2	*ccnt2*	9	↑	↓	ENSDARG00000036685
Proliferating cell nuclear antigen	*pcna*	10	↑	↓	ENSDARG00000054155
Ret proto-oncogene (probes 16, 19)	*ret*	13	↓	↑	ENSDARG00000055305
Ret proto-oncogene (probes 20, 24)	*ret*	13	↓	↓	ENSDARG00000055305
Integrin-linked kinase (probes 8, 1)	*ilk*	10	↑	↓	ENSDARG00000056964
Integrin-linked kinase (probes 6)	*ilk*	10	↑	↑	ENSDARG00000056964
Novel protein similar to vertebrate threonyl-tRNA synthetase	*tars*	18	↑	↑	ENSDARG00000075429
Ribonuclease inhibitor 1	*rnh1*	22	↓	↓	ENSDARG00000078234
Calpain 2, (m/II) large subunit, like	*capn2*	22	↑	↓	ENSDARG00000034211
Synaptopodin-2	*synp02*	7	↓	↑	ENSDARG00000079675

Genes with both significantly altered gene expression levels and DNA methylation levels (> 1.5 fold) (expression level: ↑ up regulated, ↓ down regulated; methylation level: ↑ hypermethylated, ↓ hypomethylated).

### Confirmation of the CGI tiling array data using bisulfite sequencing PCR

Based on the data obtained from the methylation tiling array, six genes from 3 different categories of hypomethylated, hypermethylated and genes showing no significant change between tumor and healthy tissue based on microarray analysis were selected for validation of the array data by bisulfite sequencing PCR (three tumor samples and three control samples). Bisulfite sequencing PCR provided a detailed analysis of the methylation status of individual CpGs within the amplified regions [30]. The *glutathione S-transferase P1 *CGI, as shown in Additional file 2, Figure S1, is un-methylated in both tumor and control samples. Following treatment with CpG methyltransferase (*SssI*) in the presence of SAM, it became fully methylated (Additional file 2, Figure S3). This artificially methylated gene was used as a positive control to confirm our semi-quantitative measurements of DNA methylation.

**Figure 9:**
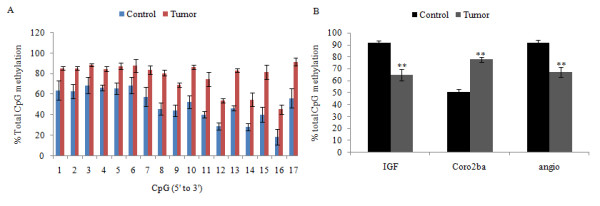
**Confirmation of the CGI tiling array using bisulfite sequencing PCR**. A. Methylation level at each CpG site was measured in 3 independent tumors and 3 independent control samples for *coro2ba *± SEM (data for *angptl1 *and *igfbp1b *is not shown; T = tumor, C = control, region: -580 to -879). B. Percentage of DNA methylation (combined for all measured CpGs) in *angptl1*, *igfbp1 *and *coro2ba *genes showed significant changes between tumor and control samples (p-value < 0.01). (*angptl1*: tumors: number of CpG sites = 21, controls: number of CpG sites = 21; *igfbp1b*:tumors: number of CpG sites = 15, controls: number of CpG sites = 15; *coro2ba*: tumors: number of CpG sites = 41, controls: number of CpG sites = 41 ± SEM).

Direct sequencing of bisulfite-treated and amplified DNA was used for measurement of average methylation percentage in a population of DNA molecules. Both normal and tumor tissues are heterogeneous in terms of molecular alterations and cell populations. As in this study DNA was extracted from a tissue with a mixture of cell populations, complete homogeneity of the sequence data was not anticipated. Therefore, the proportion of C/T was compared between samples based on well established methodology previously utilized [31-33]. Direct sequencing of bisulfite treated amplified DNA (as well as allowing the detection of partial and rare events [34]) provided an average methylation percentage at particular CpG sites in a population of DNA molecules. This is in contrast to establishment of the methylation status of a CpG in one molecule achieved by cloning prior to sequencing [31-34]. The genes selected for additional analysis using bisulfite sequencing PCR (BSP) were *coronin, actin binding protein 2ba *(*coro2ba*) (hypermethylated in HCC), *insulin-like growth factor binding protein 1b *(*igfbp1b*) and *angiopoietin-like 3 *(*angptl3*) (hypomethylated in HCC), *no-tail *(*ntla*) (no change) and *S-adenosylhomocysteine hydrolase-like 2 *(*ahcyl2*) and *inhibitor of DNA binding 2 *(*id2a*) (showed no statistically significant change). The primers and annealing temperatures used are shown in Additional file 3, Table S1. Measurement of peak area for C and T at a particular CpG site and calculation of the percentage of methylation in four-dye trace sequencing data allowed a semi-quantitative assessment of the methylation level at the specific site. To compare the methylation status of the targeted area by BSP in tumor and control, the total amount of methylation for the region was measured and compared for tumor and control. Bisulfite sequencing PCR results for *insulin-like growth factor protein 1b*, *coronin-actin binding protein 2ba *and *angiopoietin-like 3 *confirmed the changes observed from the tiling array (Figure 9, sequencing data are presented in Additional file 9, Figures S1 and S2). The three genes showing no significant difference in methylation by microarray also showed no change in methylation between tumor and normal samples by BSP.

## Discussion

To achieve a global non-biased enrichment of the methylated fragments of genomic DNA, methyl-DNA immunoprecipitation (MeDIP) combined with the novel zebrafish CpG island tiling array was used. This method enabled us to perform the first gene-specific large scale analysis and establishment of a comprehensive methylation map at CGIs of the regions 1.5 kb upstream to 1 kb downstream of the TSS in both healthy zebrafish liver and in hepatocellular carcinoma.

Regulation of transcription is a complex procedure. It is partly accomplished by formation of nucleosomes [35] and by modulating the binding of regulatory factors and the transcription complex to transcriptional response elements, both directly and indirectly [1]. Epigenetic mechanisms such as chromatin modifications, RNA interference (RNAi) and DNA methylation are key modulators of transcription through different mechanisms, such as prohibiting transcription factor access to their binding sites and affecting the formation of nucleosomes [12,35,36].

The methylation profiles of cancer cells are extensively distorted [11]. Hypomethylation in tumors is associated with transcriptional activation of previously suppressed genes and is a hallmark of tumorigenesis [1,11], where active genes are un-methylated with hyperacetylated histones [12]. As shown in Table 1A, GO terms associated with DNA binding and transcription regulation were significantly over-represented amongst genes hypomethylated in HCC samples. Genes including *histone deacetylase 4, DNA (cytosine-5-)-methyltransferase 6 *and *lysine-specific demethylase 4A *showed significant decreases in their methylation levels in HCC samples in comparison to healthy liver.

Comparison of the normal hepatic gene methylation pattern between human liver, presented by Archer et al [37], and zebrafish liver in this study showed a low correlation (r^2 ^= 0.187) implying lack of conservation of methylation patterns between human and zebrafish. However, our comparisons showed that, in zebrafish and human HCC tumors, similar gene families and genes involved in shared pathways are altered in terms of methylation. For example, zebrafish HCC samples showed methylation changes in genes involved in proliferation, cell cycle, metastasis, apoptosis, energy production, adhesion, stress, DNA binding and regulation of transcription (Table 1A, B and Additional file 6, Tables S2 and S3), similar to the biological processes that contain genes with altered methylation in human HCC [2]. Several other genes from families such as ABCA, CHST, DHX, KCTD, MEGF, MYO, NPY, RNF and TBCID were found to be hypermethylated in both zebrafish and human HCC [38]. Ingenuity Pathway Analysis indicated that the genes with altered methylation in zebrafish hepatocellular carcinoma were associated with biological functions such as cell death, cell morphology, inflammatory response, DNA repair and replication and induced molecules involved in cancer formation such as *c-jun, shc *and *pka*. These functions and molecules are commonly altered in human cancers. We have associated changes in methylation of these particular genes with changes in gene expression during tumorigenesis. The changes in methylation levels of these particular genes and pathways could be directly or indirectly linked to their altered expression levels during tumorigenesis.

In our study, GO terms associated with cell proliferation were significantly over-represented in the list of hypomethylated genes in tumors which is particularly relevant since an imbalance between regulation of cellular proliferation pathways and cell death by apoptosis can promote the development of tumors [39]. Anti-apoptotic genes, such as *BCL-2*, and their regulators, are often over expressed in human tumors [40,41]. Our results showed a significant decrease in the methylation of a positive regulator of the Bcl-2 protein, *bcl-2 associated athanogene 5 *(*baga5*) gene [40]. Changes in anti-apoptotic pathways in zebrafish HCC were concurrent with changes in pathways of proliferation. Insulin like growth factors (IGF) and insulin like growth factor binding proteins (IGFBPs) play important roles in organizing cell proliferation, apoptosis and differentiation and are commonly deregulated in human tumors [42]. In zebrafish HCC, genes for several insulin growth factor binding proteins (IGFBPs) such as *igfbp2b *were significantly hypomethylated. The promoter region of the human *IGFBP-2 *gene is rich in CpGs and lacks a TATA box [43]. It is therefore plausible that methylation plays an important role in regulating the expression of this gene. Multiple complex IGF-dependent and independent biological functions influenced by the tissue type and pathological status have been identified for IGFBPs [42,43]. An increased level of IGFBP-2 protein has been reported in liver tissues and serum during human malignancy [42,43] with a positive correlation to the malignancy status of the tumor [43]. In contrast to its normal role as a negative regulator of growth, increased levels of IGFBPs in tumors have been linked to enhanced proliferation, partly as a response to androgens and hypoxia-inducible factor-1 (HIF-1) protein. Thus, anaerobic conditions as well as IGF, can result in increased amount of HIF-1 [44]. A lack of vascular supply at the early stages of tumorigenesis in highly proliferating tumor cells results in hypoxia [44]. Under hypoxic circumstances glycolysis becomes the dominant pathway for energy production in tumors and glycolytic enzymes are induced. Associated with this, the HIF-1 protein, expressed in an anaerobic environment, initiates the transcription of several genes involved in stress and glycolysis as well as *IGFBP2 *[43,44]. This is in accord with our finding that GO terms related to glycolysis and hypoxia pathways were more prevalent in the list of hypomethylated genes in HCC samples. Glycolytic enzymes such as enolase 2 (ENO2) and hexokinase 2 showed significant decreases in methylation of their genes in HCC samples, indicating a potential increase in expression. Increased expression of enolases such as *ENO1 *and *2*, as a response to hypoxia and HIF-1, has been reported in human HCC [45]. As well as its function in energy production, ENO1 has been associated with enhanced proliferation in HCC [45]. Therefore, based on the functions of genes whose methylation was significantly altered in HCC samples it appears that there is a link between induction of IGF, IGFBPs, HIF, anti-apoptotic and glycolytic pathways [43,44,46]. This is similar to findings previously reported on gene expression in human HCC implying that differential methylation is at least partially causative of differential gene expression in HCC.

## Conclusion

In this paper we described the development of a zebrafish CGI tiling array and showed that in combination with the MeDIP technique, it can be used to detect and profile the methylation status of specific genes. We used this method to provide a comprehensive profiling of zebrafish liver methylation as well as establishing the methylation changes observed in HCC.

The methylation alterations detected can help to explain some of the changes in gene expression in HCC. There are striking similarities between the pathways disrupted by aberrant gene expression and methylation, both within and between human and zebrafish HCC. Achieving a better understanding of genetic and epigenetic regulation in the zebrafish will increase the confidence regarding the use of zebrafish as a convenient model for human disease.

## Methods

All chemicals were obtained from Sigma-Aldrich, Poole, Dorset, UK, unless otherwise stated.

### Measurement of genome wide DNA methylation by HPLC

An AKTA Explorer 10 with P900 pump, automatic UV detector (Amersham Biosciences), APEX ODS C18 column, 250 × 4.6 mm i.d., 5 μm particle size (Waters HPLC Ltd, UK, Phenomenex, UK); and grade column (Phenomenex, UK) were used. Following purification of DNA using Qiagen DNA/RNA purification kit (Qiagen, Ltd, West Sussex, UK), percentage of global DNA methylation was quantified according to the method of Ramsahoye [47].

### Experimental procedure and sample preparation

Methyl-DNA immunoprecipitation in combination with a CpG island tiling array was used for profiling methylation patterns in un-treated zebrafish liver and hepatocellular carcinoma (HCC) samples. The chemical treatment procedure and doses used for generating zebrafish tumors are described by Zhan et al [48]. The exposures were carried out at the National University of Singapore and the experimental procedure was approved by its Institutional Animal Care and Use Committee. Briefly, three-week-old zebrafish fry were treated with 0.75 ppm 7, 12-dimethylbenz[α]anthracene (DMBA) or dimethyl sulfoxide (DMSO, vehicle control) for 24 h and the treatment was repeated once 2 weeks later for another 24 hours with 1.25 ppm DMBA or DMSO. Treated fish were rinsed three times in fresh water and transferred into new tanks for maintenance. Fish were sampled 6-10 months after DMBA exposure. The tumor samples used for the present study were all larger than 3 mm in diameter. Liver tumors were sampled for histopathological diagnosis (histopathology images of HCC and healthy zebrafish liver are presented in Additional file 10, Figure S1 and histopathology procedures and criteria used for recognizing HCC are explained in detail in a previous publication 23). Briefly, criteria for classification of liver neoplasms were based on nuclear factors described by Goodman et al [49]. In this study, healthy male livers (n = 4) from the vehicle control exposure (n = 4), hepatocellular carcinoma from the DMBA exposure (n = 4, male) and positive and negative controls artificially generated from the vehicle control exposure (detailed below) were used for MeDIP and immunoprecipitated DNA was labeled with fluorescent Cy5-dCTP. A non-immunoprecipitated genomic DNA pool was used as a reference and was labeled with fluorescent Cy3-dCTP.

### Design of the 4 × 44 k format CpG island (1.5 kb downstream to 1 kb upstream of TSS) zebrafish tiling array

Probes were designed by Genotypic (Genotypic Technology, Bangalore, India) and were synthesized and printed by Agilent (Agilent technologies, Berkshire, UK). The criteria described by Gardiner-Garden and Frommer [50] for prediction of CpG islands in vertebrates were used to predict CpG islands in zebrafish, with minor modifications. The zebrafish genome sequence was derived from the Ensembl data base (version 56, genome build zv8, genome build date April 2009). For each gene, the region 1.5 kb upstream to 1 kb downstream of predicted transcription start sites (TSS) was found, using the 5' end of each transcript as the putative TSS. Repetitive sequences were masked and the Emboss CpG prediction tool (European Molecular Biology Laboratory-EBI) identified 9,192 genes containing 14,507 CpG islands. From the preliminary list of genes (9,192), 60-mer probes were designed with an average spacing of 25 bp where possible. Due to the requirements for probe specificity, probes could not be constructed for all predicted CGIs. Probes with multiple BLAST hits on the zebrafish genome and with identical 55 bp alignments were removed. Finally, 43,960 probes were designed for 7,903 CGIs in 6,024 genes. Slides were printed containing 45,220 features; 1,227 Agilent control features, 33 blank features and 43,960 probes. The array design was named Agilent Birmingham *D.rerio *025794 45220v1 and is available from ArrayExpress under accession A-MEXP-1813. Lists of genes with predicted CGIs and the detailed criteria used for prediction of CGIs are presented in Additional file 5, Table S1. Probes were functionally annotated from the zebrafish genome and additional gene ontology (GO) terms were found using Blast2GO [51].

The "Core Analysis" function included in Ingenuity Pathway Analysis (IPA) (Ingenuity Systems Inc, USA) [52] was used to aid interpretation of the hypo- and hyper-methylated (fold change > 1.5) genes found by comparison of zebrafish HCC with healthy liver. A Benjamini and Hochberg multiple testing correction was employed to determine significant enrichment of annotation with biological functions and canonical pathways among these gene sets.

### Positive and Negative Controls

CpG methyltransferase (New England Biolabs, U.S.A) was used to generate artificially methylated DNA from zebrafish liver genomic DNA using *S*-adenosyl methionine according to the manufacturer's protocol. Briefly, 10×NEBuffer 2 (2 μl), S-adenosyl methionine (160 μM), genomic DNA (1 μg) and *Sss*I methylase (40 U/μl; New England Biolabs, U.S.A) were mixed in a total volume of 20 μl. The sample was incubated at 37°C for 4 h followed by incubation at 65°C for 20 min for inactivation of the enzyme.

A negative control for the array was generated by PCR amplification of sonicated zebrafish liver genomic DNA. DNA samples were amplified using GenomePlex Complete Whole Genome Amplification (WGA) Kit (Sigma-Aldrich, Poole, Dorset, U.K). PCR amplification removes DNA methylation, resulting in an artificially un-methylated DNA. Positive and negative control samples were purified (Qiagen) prior to use in methyl-DNA immunoprecipitation.

### Methyl-DNA immunoprecipitation (MeDIP)

DNA from tumor, control, SAM-treated DNA (positive control) and amplified DNA (negative control) were dissolved in TE buffer (6 μg in 300 μl) and fragmented to 200 bp-1 Kb using a sonicator (SONICS Vibra Cell, 100 watt, 3 × 10 s with 35 s intervals on ice with 20% amplitude). Sizes of the generated fragments were checked on a 1% agarose gel.

Methylated fragments of DNA were separated from the un-methylated fragments using MagMeDIP kit (Diagenode, Belgium) according to the manufacturer's instructions. DNA sample (1 μg) was used in each immunoprecipitation. To avoid amplification bias, five aliquots of each sample were immunoprecipitated and combined. Samples were re-purified by phenol/chloroform/isoamyl alcohol extraction and precipitated with ethanol and glycogen. DNA pellets were air dried and stored at -80°C until subsequent use for labeling.

### Sample labeling and hybridization to CGI (1.5 kb downstream to 1 kb upstream of TSS) zebrafish tiling array

The Agilent genomic DNA enzymatic labeling kit (Agilent technologies, Berkshire, UK) and the protocol provided were used for labeling the immunoprecipitated DNA with the fluorophore Cy-5 and zebrafish genomic DNA with the fluorophore Cy-3. The specific activities of the labeled samples were checked using a NanoDrop spectrophotometer. Cy3-labeled genomic DNA samples were pooled. For each hybridization, equal amounts (80 pmol incorporated) of Cy-3 labeled genomic DNA and Cy-5 labeled immunoprecipitated DNA were mixed. Microarray hybridization was performed using an Agilent Oligo aCGH/Chip on chip hybridization kit according to the manufacturer's protocol. Briefly, the hybridization mixes were loaded onto 4 × 44 K format slides (Agilent Birmingham *D.rerio *025794 45220v1) hybridized overnight, washed, stabilized and dried. The dried slides were scanned using an Axon 4000B laser scanner (Molecular Devices, Wokingham, UK).

### Statistical analysis

MIAME-compliant raw microarray data were submitted to ArrayExpress at EMBL-EBI and can be found under accession E-MTAB-209. GeneSpring v7.2 (Agilent) was used for analyzing the data. Data flagged as present in at least 4 of the 10 samples were used for analyses. The Cy-5 test signal was divided by the control signal (genomic pool) and each microarray was normalized to the mean of the control group. Data with low raw intensity (less than 25) or standard deviation greater than 1.4 between biological replicates were removed from the analyses. Lists of hypo- and hyper- methylated genes were generated using 1.5 fold change cut-offs and parametric Welch t-tests between tumor and healthy groups (p-value <0.05) on the normalized data.

### DNA methylation analysis using bisulfite sequencing PCR

The MethPrimer data base (MethPrimer - Li Lab, UCSF) was used to design BSP primers (primer sequences and annealing temperatures used are listed in Additional file 1, Table S1). An artificially methylated positive control was used for assessing complete conversion of un-methylated cytosine to uracil after treatment with bisulfite and methylation of all CpG dinucleotides. The EZ DNA Methylation kit (Cambridge Biosciences, UK) was used for bisulfite conversion according to manufacturer's protocol. Briefly, for each sample, 500 ng of genomic DNA was bisulfite treated then amplified using Zymo *Taq *DNA polymerase (Cambridge Biosciences, UK). The PCR products were analyzed via DNA gel electrophoresis followed by sequencing using an ABI3730 DNA analyzer.

## Authors' contributions

LM, TDW and JKC designed the experiment and analyzed the data. LM performed the experiments and drafted the manuscript. TDW and JKC supervised the project. JKC provided financial support. HZ generated carcinogen-induced zebrafish tumors. ZG supervised chemical carcinogenesis experiments and provided zebrafish gene expression data. All authors read and approved the final manuscript.

## Supplementary Material

Additional file 1 **Table S1. Primers used in bisulfite sequencing PCR**. List of bisulfite sequencing primers (14 genes, 15 regions) used for optimization of the immunoprecipitation method for HCC and healthy zebrafish liver samples are shown in this table. Annealing temperatures, product sizes and Ensembl gene IDs are shown. Click here for file

Additional file 2 **Optimization of bisulfite sequencing PCR**. MeDIP validation (Figures S1 and S2). Bisulfite sequencing of *glutathione S-transferase P1 *gene (*gstp*1) (Figure S1) showed complete un-methylation of the CpG dinucleotides. Bisulfite sequencing of the *no-tail *(*ntla*) gene showed complete methylation of the CpG dinucleotides (Figure S2). (Sequencing was performed on 4 independent samples; two tumor samples and two control samples, arrows: cytosine located at CpG dinucleotide site. Red peak: T, Blue peak: C) Validation of bisulfite sequencing PCR (Figure S3). *Glutathione S-transferase P1 *gene is un-methylated in both tumor and control. Treatment with CpG methyltransferase (*Sss I*) in the presence of *S*-adenosylmethionine (SAM) results in methylation of all CpG dinucleotides and protection against conversion of 5-mC to U after treatment with bisulfite. Successful bisulfite treatment and sequencing of the positive control was established by observing a single C peak at the CpG dinucleotide positions. (Two independent tumor samples, two independent control samples). 1 = SAM positive control, 2 = control, 3 = control, 4 = tumor, 5 = tumor. Arrows: cytosine located at CpG dinucleotide site. Click here for file

Additional file 3 **Table S1. Array data derived from comparison of methylation level between HCC and healthy zebrafish liver were confirmed using bisulfite sequencing PCR**. Primer sequences, targeted CpG islands, and annealing temperatures are shown in this table. Click here for file

Additional file 4 **Figure S1. Agarose gel electrophoresis image of methyl-DNA immunoprecipitated (MeDIP) bisulfite treated *ntla *and *gstp1***. The methylated *ntla *(a) gene is enriched after immunoprecipitation, whereas the un-methylated *gstp1 *(b) gene is not enriched. Click here for file

Additional file 5 **Table S1. CpG island prediction**. A list of genes with predicted CGIs, number of CGIs for each chromosome and location on the chromosome are presented in additional file 5, Table S1. Click here for file

Additional file 6 **Full list of array data**. Table S1: Fluorescence signals of positive, artificially-methylated and negative, artificially un-methylated immunoprecipitated controls relative to that of genomic DNA. Table S2: Genes hypomethylated in tumor samples (> 1.5 fold). Table S3: Genes hypermethylated in tumor samples (> 1.5 fold). Table S4: List of gene methylation levels in control samples. Click here for file

Additional file 7 **Ingenuity Pathway Analysis of significantly altered genes (fold change > 1.5 fold) in zebrafish hepatocellular carcinoma compared to control achieved from MeDIP-tiling array**. Table S1: Functional categories enriched among hypermethylated genes with FDR <5%. The categories, functional annotations and molecules identified in each category are also presented. Table S2: Functional categories enriched among hypomethylated genes with FDR <5%. The categories, functional annotations and molecules identified in each category are also presented. Click here for file

Additional file 8 **Table S1. List of genes (194) investigated for comparison of DNA methylation level and gene expression level in zebrafish HCC and healthy zebrafish liver**. Genes highlighted in blue (22) have significant changes in both gene expression and DNA methylation. Click here for file

Additional file 9 **Confirmation of the tiling array data using bisulfite sequencing PCR**. Figure S1: Shows relative increase in methylation of *coronin-actin binding protein 2ba *in tumor compared with control. Figure S2: Relative decrease in methylation of *angiopoietin-like 3 *in tumor compared with control. Figure S3: Relative decrease in methylation of *insulin-like growth factor binding protein 1b *in tumor compared with control. (1, 2 and 3: three independent controls, 4, 5 and 6: three independent tumors). Click here for file

Additional file 10 **Figure S1. Histopathology images of zebrafish HCC and healthy zebrafish liver**. A. Zebrafish liver section with hepatocellular carcinoma (HCC) invasion into surrounding normal liver tissue. HCC and surrounding normal tissue are labeled. B. Enlarged image of the section indicated with a box in image A showing the boundary between HCC and normal tissue. C. Normal control liver section. Normal zebrafish hepatocytes are typically organized in two-cell thick plates and are regular throughout the whole liver. Carcinomas lose this plate architecture completely and are reorganized in typical patterns, such as a trabecular pattern with several-cell thick irregular trabeculae, a glandular pattern with a central clear space surrounded by one-cell layer neoplastic hepatocytes, and a large sheet of neoplastic cells without any recognizable pattern. Carcinoma cells are cuboidal with centrally localized nuclei of variable sizes. Click here for file
