# Supplementary figures and images for: Comprehensive profiling of zebrafish hepatic proximal promoter CpG island methylation and its modification during chemical carcinogenesis
Source: BMC Genomics. 2011 Jan 4;12:3. doi: 10.1186/1471-2164-12-3 (PMC3027158; doi:10.1186/1471-2164-12-3)

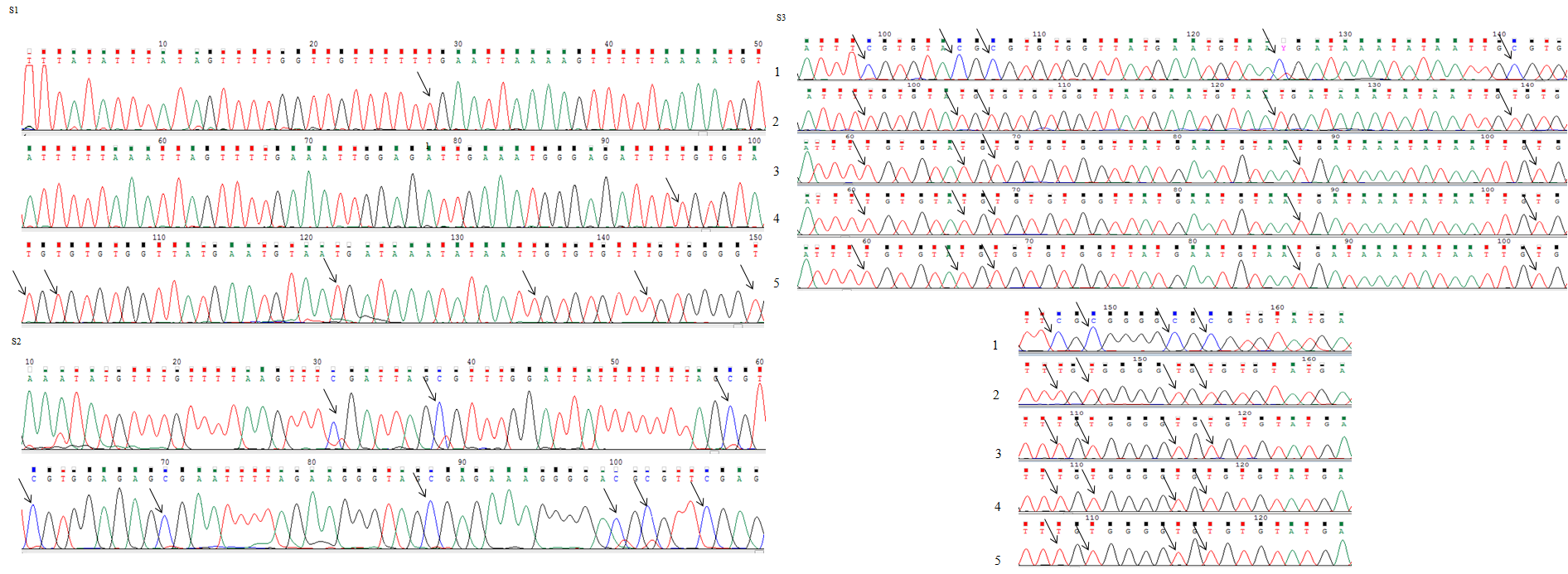

Supplement: Additional file 2 — Optimization of bisulfite sequencing PCR. MeDIP validation (Figures S1 and S2). Bisulfite sequencing of glutathione S-transferase P1 gene (gstp1) (Figure S1) showed complete un-methylation of the CpG dinucleotides. Bisulfite sequencing of the no-tail (ntla) gene showed complete methylation of the CpG dinucleotides (Figure S2). (Sequencing was performed on 4 independent samples; two tumor samples and two control samples, arrows: cytosine located at CpG dinucleotide site. Red peak: T, Blue peak: C) Validation of bisulfite sequencing PCR (Figure S3). Glutathione S-transferase P1 gene is un-methylated in both tumor and control. Treatment with CpG methyltransferase (Sss I) in the presence of S-adenosylmethionine (SAM) results in methylation of all CpG dinucleotides and protection against conversion of 5-mC to U after treatment with bisulfite. Successful bisulfite treatment and sequencing of the positive control was established by observing a single C peak at the CpG dinucleotide positions. (Two independent tumor samples, two independent control samples). 1 = SAM positive control, 2 = control, 3 = control, 4 = tumor, 5 = tumor. Arrows: cytosine located at CpG dinucleotide site. [file 1471-2164-12-3-S2.PNG]

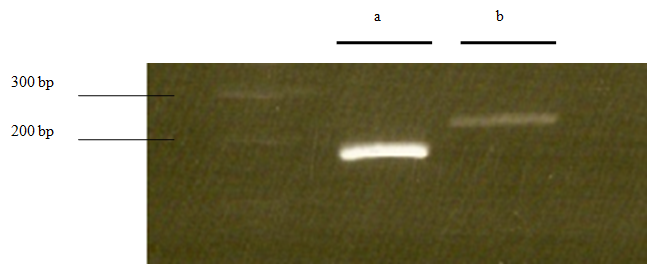

Supplement: Additional file 4 — Figure S1. Agarose gel electrophoresis image of methyl-DNA immunoprecipitated (MeDIP) bisulfite treated ntla and gstp1. The methylated ntla (a) gene is enriched after immunoprecipitation, whereas the un-methylated gstp1 (b) gene is not enriched. [file 1471-2164-12-3-S4.PNG]

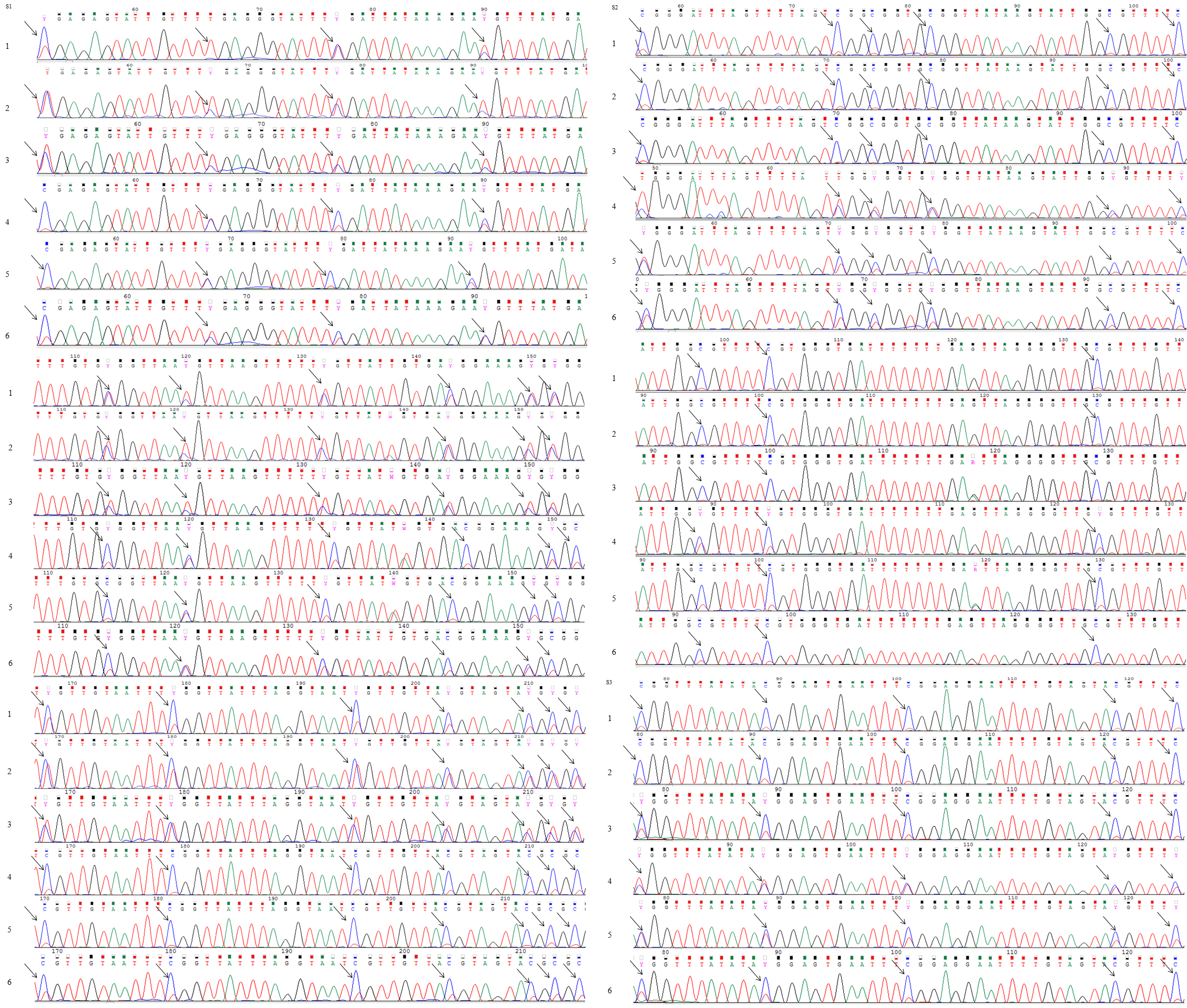

Supplement: Additional file 9 — Confirmation of the tiling array data using bisulfite sequencing PCR. Figure S1: Shows relative increase in methylation of coronin-actin binding protein 2ba in tumor compared with control. Figure S2: Relative decrease in methylation of angiopoietin-like 3 in tumor compared with control. Figure S3: Relative decrease in methylation of insulin-like growth factor binding protein 1b in tumor compared with control. (1, 2 and 3: three independent controls, 4, 5 and 6: three independent tumors). [file 1471-2164-12-3-S9.PNG]

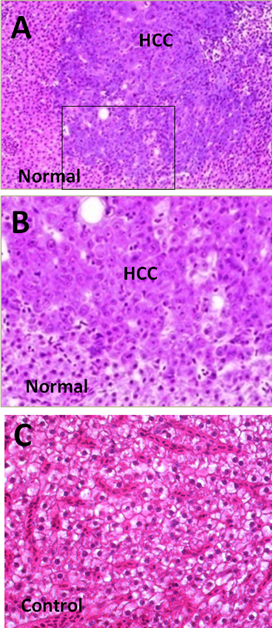

Supplement: Additional file 10 — Figure S1. Histopathology images of zebrafish HCC and healthy zebrafish liver. A. Zebrafish liver section with hepatocellular carcinoma (HCC) invasion into surrounding normal liver tissue. HCC and surrounding normal tissue are labeled. B. Enlarged image of the section indicated with a box in image A showing the boundary between HCC and normal tissue. C. Normal control liver section. Normal zebrafish hepatocytes are typically organized in two-cell thick plates and are regular throughout the whole liver. Carcinomas lose this plate architecture completely and are reorganized in typical patterns, such as a trabecular pattern with several-cell thick irregular trabeculae, a glandular pattern with a central clear space surrounded by one-cell layer neoplastic hepatocytes, and a large sheet of neoplastic cells without any recognizable pattern. Carcinoma cells are cuboidal with centrally localized nuclei of variable sizes. [file 1471-2164-12-3-S10.PNG]
